# Interleukin-10 Protection against Lipopolysaccharide-Induced Neuro-Inflammation and Neurotoxicity in Ventral Mesencephalic Cultures

**DOI:** 10.3390/ijms17010025

**Published:** 2015-12-28

**Authors:** Yan Zhu, Xiao Chen, Zhan Liu, Yu-Ping Peng, Yi-Hua Qiu

**Affiliations:** 1 School of Biological & Basic Medical Sciences, Soochow University, 199 Renai Road, Suzhou 215123, China; zhuyan@ntu.edu.cn; 2 Department of Physiology, School of Medicine, and Co-innovation Center of Neuroregeneration, Nantong University, 19 Qixiu Road, Nantong 226001, China; 13160049@yjs.ntu.edu.cn (X.C.); liuzhan@ntu.edu.cn (Z.L.)

**Keywords:** IL-10, LPS, Parkinson’s disease, dopaminergic neurons, microglia, astrocytes

## Abstract

Interleukin (IL)-10, an anti-inflammatory cytokine, is expressed in the brain and can inhibit microglial activation. Herein, we utilized lipopolysaccharide (LPS)-induced inflammatory Parkinson’s disease (PD) cell model to determine whether microglia and astrocytes are necessary targets for IL-10 neuroprotection. Primary ventral mesencephalic (VM) cultures with different composition of neurons, microglia and astrocytes were prepared. The cells were exposed to IL-10 (15, 50 or 150 ng/mL) 1 h prior to LPS (50 ng/mL) treatment. LPS induced dopaminergic and non-dopaminergic neuronal loss in VM cultures, VM neuron-enriched cultures, and neuron-microglia co-cultures, but not in neuron-astrocyte co-cultures. IL-10 reduced LPS-induced neuronal loss particularly in single VM neuron cultures. Pro-inflammatory mediators (TNF-α, IL-1β, inducible nitric oxide synthase and cyclooxygenase-2) were upregulated in both neuron-microglia and neuron-astrocyte co-cultures by LPS. In contrast, neurotrophic factors (brain-derived neurotrophic factor, insulin-like growth factor-1 or glial cell-derived neurotrophic factor) were downregulated in neuron-microglia co-cultures, but upregulated in neuron-astrocyte co-cultures by LPS. IL-10 reduced both the increase in production of the pro-inflammatory mediators and the decrease in production of the neurotrophic factors induced by LPS. These results suggest that astrocytes can balance LPS neurotoxicity by releasing more neurotrophic factors and that IL-10 exerts neuroprotective property by an extensive action including direct on neurons and indirect via inhibiting microglial activation.

## 1. Introduction

Neuro-inflammatory process has been associated with most neurodegenerative diseases including Parkinson’s disease (PD), Alzheimer’s disease (AD), multiple sclerosis (MS) and Huntington’s disease [1]. Neuro-inflammation is characterized by the activation of brain glial cells, primarily microglia and astrocytes that release various soluble factors that include free radicals (reactive oxygen species (ROS) and reactive nitrogen species (RNS)), cytokines, and lipid metabolites [2]. The majority of these glia-derived factors are pro-inflammatory and neurotoxic and are particularly deleterious to oxidative damage-vulnerable nigral dopaminergic neurons [2]. In addition to the increased pro-inflammatory mediators in the brain in neurodegenerative diseases, neurotrophic factors, such as brain-derived neurotrophic factor (BDNF), glial cell-derived neurotrophic factor (GDNF) and insulin-like growth factor (IGF)-1, are impaired in these diseases [3,4,5,6,7,8,9,10,11]. To further show an involvement of neuro-inflammation in neurodegenerative diseases, we employed an *in vitro* inflammatory PD model induced by lipopolysaccharide (LPS) in this study.

LPS, an endotoxin found in outer membrane of gram-negative bacteria, is a potent stimulator of both peripheral immune cells (macrophages and monocytes) and brain glia (microglia and astrocytes) and causes their release of various immunoregulatory and pro-inflammatory cytokines and free radicals [12,13,14,15]. The important pro-inflammatory mediators produced in response to LPS stimulation include tumor necrosis factor (TNF)-α, interleukin (IL)-1β, ROS (namely hydrogen peroxide(H_2_O_2_)), RNS (*i.e.*, nitric oxide(NO)) that is induced by inducible nitric oxide synthase (iNOS), and prostaglandin E2 that is induced by cyclooxygenase (COX)-2 [16,17,18]. Overproduction of the pro-inflammatory factors leads to neuronal death in the brain [19,20,21]. Although LPS is an effectively non-specific stimulator of glial cells in the brain, it has been commonly used to induce a model of PD to show an interaction between neuro-inflammation and neurodegeneration [22,23,24]. PD is characterized pathologically by a selective loss of dopaminergic neurons that express tyrosine hydroxylase (TH) in the nigrostriatal system. Recently, accumulating evidence has shown that neuro-inflammation is a major component of pathophysiology of PD and contributes to cascade of events leading to dopaminergic neuronal degeneration [25]. Characteristics of various *in vitro* and *in vivo* LPS models of PD have been described in details [2]. However, it remains controversial whether LPS directly damages to dopaminergic neurons. Accordingly, we utilized primary ventral mesencephalic (VM) cultures with different composition of neurons, microglia and astrocytes to identify roles of microglia and astrocytes in LPS-induced dopaminergic neurodegeneration.

Since neuro-inflammation is involved in PD occurrence and development, an anti-inflammatory treatment is promising to alleviate PD pathology and symptoms. Interleukin (IL)-10, a pleiotropic cytokine, is endogenously produced by activated immune cells including T cells, B cells and macrophages [26]. It mainly drives a regulation of a variety of anti-inflammatory processes [27]. In the brain, IL-10 is expressed by monocytes, astrocytes and microglia [28] as well as by neurons [29]. Recent research has found that IL-10 expression is downregulated in the substantia nigra of patients with PD [29]. Osmotic pump infusion of IL-10 into the substantia nigra protects against LPS-induced cell death of dopaminergic neurons, with a corresponding decrease in the number of activated microglia, suggesting that the reduction in microglia-mediated release of inflammatory mediators may contribute to the anti-inflammatory effect of IL-10 [30]. However, it is unclear whether IL-10 directly protects dopaminergic neurons against LPS toxicity. Herein, firstly we identify that LPS also exerts a direct toxicity to neurons; secondly, we establish that IL-10 reduces LPS-induced neuronal loss in either the presence or the absence of glial cells; and lastly, we demonstrate that IL-10 inhibits LPS-induced glial activation by downregulation of pro-inflammatory mediators and upregulation of neurotrophic factors. A potential therapeutic strategy for PD is to limit development of inflammatory response [25,31]. Our present study provides a new cue for IL-10 alleviation of PD neurodegeneration by its anti-inflammatory property.

## 2. Results

### 2.1. IL-10 Reduces LPS-Induced TH^+^NeuN^+^ and TH^−^NeuN^+^ Neuronal Loss in VM Cultures, VM Neuron-Enriched Cultures, or Neuron-Microglia Co-Cultures

**Figure 1:**
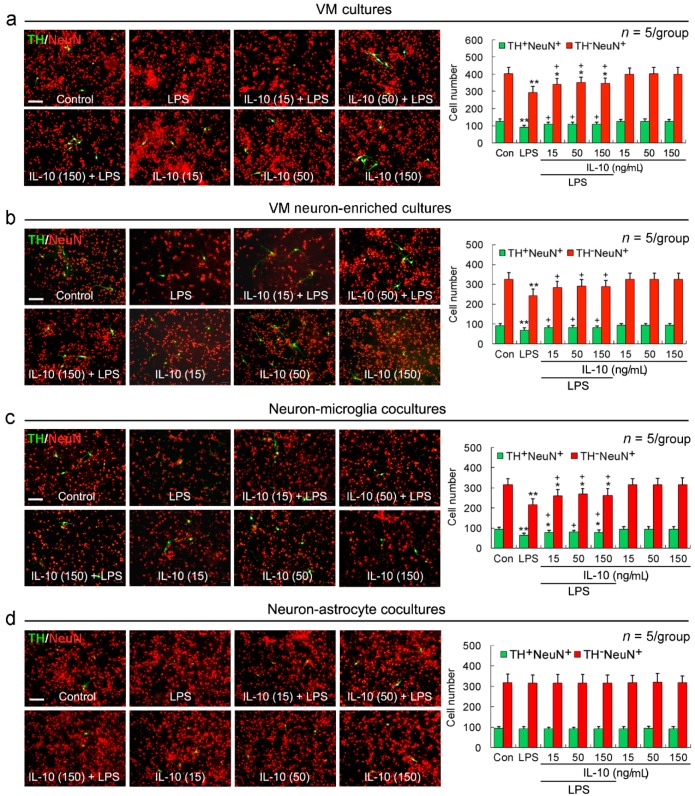
IL-10 reduces LPS-induced TH^+^NeuN^+^ and TH^−^NeuN^+^ neuronal loss in VM cultures, VM neuron-enriched cultures, or neuron–microglia co-cultures. The different cell-composition cultures were pretreated with IL-10 (15, 50 or 150 ng/mL) for 1 h and then exposed to LPS (50 ng/mL), which were incubated for 24 h. Cells were fixed and stained for TH and NeuN as described in Experimental Section. (**a**–**d**) **Left panels** are representative images indicating TH/NeuN-immunoreactive cells in the different cell-composition cultures with various treatments, and **right panels** are statistical graphs corresponding to the left panels. The number of TH^+^NeuN^+^ cells was counted in 25 visual fields on each coverslip and reported as a sum, and the number of TH^−^NeuN^+^ cells was counted in five visual fields on each coverslip and reported as an average. The data are mean and standard deviation of five independent experiments. * *p* < 0.05, ** *p* < 0.01, *versus* control (Con); ^+^ *p* < 0.05, *versus* LPS.IL-10 (15): IL-10 (15 ng/mL); IL-10 (50): IL-10 (50 ng/mL); IL-10 (150): IL-10 (150 ng/mL). Scale bar = 100 μm.

To show cell targets of IL-10 neuroprotection, we employed the four different cell-composition cultures, VM cultures, VM neuron-enriched cultures, neuron-microglia co-cultures, and neuron-astrocyte co-cultures. LPS induced a decrease in the number of both TH^+^NeuN^+^ and TH^−^NeuN^+^ cells in VM cultures (Figure 1a), VM neuron-enriched cultures (Figure 1b), or neuron-microglia co-cultures (Figure 1c), but not in neuron-astrocyte co-cultures (Figure 1d), when compared with untreated control. The combined treatment with IL-10 (15, 50, or 150 ng/mL) and LPS (50 ng/mL) significantly increased both TH^+^NeuN^+^ and TH^−^NeuN^+^ cell number, with respect to LPS treatment alone (Figure 1). IL-10 exposure alone did not alter either TH^+^NeuN^+^ or TH^−^NeuN^+^ cell number in all the four cultures relative to untreated control (Figure 1).

### 2.2. LPS Increases both Neuronal Loss and NO and H_2_O_2_ Production in a Concentration-Dependent Manner, and These LPS Effects Are Alleviated By IL-10

**Figure 2:**
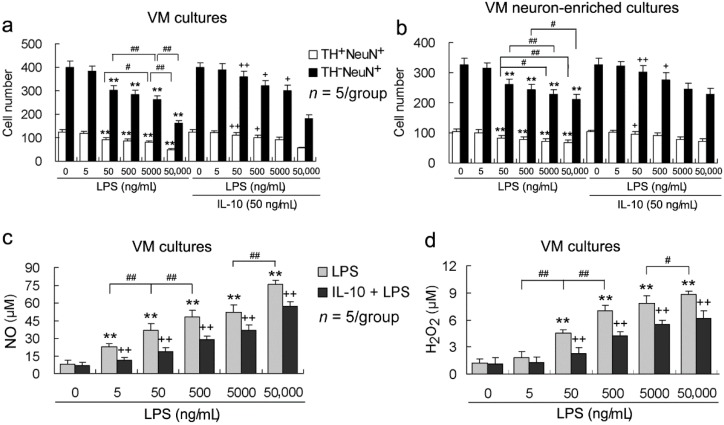
LPS increases both neuronal loss and NO and H_2_O_2_ production in a concentration-dependent manner, and these LPS effects are alleviated by IL-10. The VM cultures or VM neuron-enriched cultures were pretreated with IL-10 (50 ng/mL) for 1 h and then exposed to the various concentrations of LPS, which were incubated for 24 h. (**a,b**) The TH^+^NeuN^+^ and TH^−^NeuN^+^ cell numbers were counted and reported as described in Figure 1; (**c,d**) For assessment of NO and H_2_O_2_ release levels, the culture supernatants were collected and assayed as described in Experimental Section. The data are mean and standard deviation of five separate experiments. ** *p* < 0.01, *versus* control without any treatment; ^+^ *p* < 0.05, ^++^ *p* < 0.01, *versus* corresponding concentration of LPS;^#^ *p* < 0.05, ^##^ *p* < 0.01, between the different concentrations of LPS.

To confirm a concentration-dependent effect of LPS on neurons and microglia, we determined dopaminergic and non-dopaminergic neuronal numbers as well as NO and H_2_O_2_ production in the stimulation with 5, 50, 500, 5000 or 50,000 ng/mL of LPS in VM cultures and VM neuron-enriched cultures. Both TH^+^NeuN^+^ and TH^−^NeuN^+^ cell numbers were decreased by LPS of 50, 500, 5000 or 50,000 ng/mL (but not 5 ng/mL) in both VM cultures and VM neuron-enriched cultures (Figure 2a,b). These LPS effects were concentration-dependent (Figure 2a,b). Significantly, IL-10 (50 ng/mL) reduced the neurotoxic roles of 50, 500 or 5000 ng/mL (but not 50,000 ng/mL) of LPS (Figure 2a,b). In addition, LPS (5 or 50 to 50,000 ng/mL) induced a rapid and dose-dependent accumulation of NO and H_2_O_2_ in the culture medium of both VM cultures and VM neuron-enriched cultures (Figure 2c,d). IL-10 (50 ng/mL) significantly reduced both NO and H_2_O_2_ release induced by various concentrations of LPS (Figure 2c,d).

### 2.3. IL-10 Reduces LPS-Induced Upregulation of Pro-Inflammatory Mediators in All the Four Different Cell-Composition Cultures

**Figure 3:**
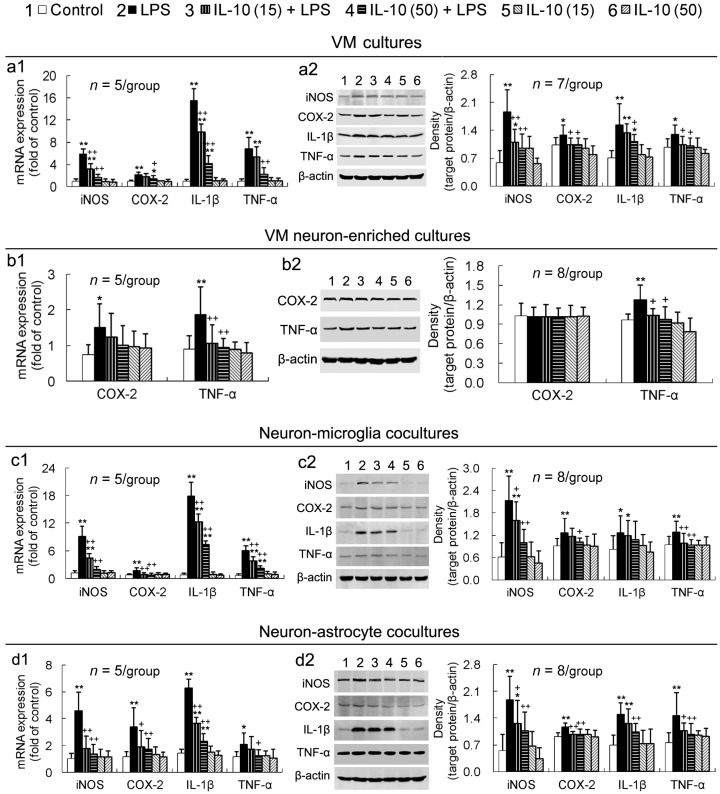
IL-10 reduces LPS-induced upregulation of pro-inflammatory mediators in all the four different cell-composition cultures. The cultures were pretreated with IL-10 (15 or 50 ng/mL) for 1 h and then LPS (50 ng/mL) was added to the cultures, which were incubated for 24 h. Total RNA and protein in the cells were extracted and subjected to real-time PCR quantification and Western blot assay for expression of the pro-inflammatory mediator genes (**a1**,**b1**,**c1**,**d1**) and proteins(**a2**,**b2**,**c2**,**d2**), respectively. * *p* < 0.05, ** *p* < 0.01, *versus* control; ^+^ *p* < 0.05, ^++^ *p* < 0.01 *versus* LPS. IL-10 (15): IL-10 (15 ng/mL); IL-10 (50): IL-10 (50 ng/mL).

In VM cultures, the pro-inflammatory mediators, iNOS, COX-2, IL-1β and TNF-α, were upregulated at both mRNA and protein expression levels by LPS; IL-10 (15 or 50 ng/mL) significantly reduced the upregulated expression of all the pro-inflammatory mediators induced by LPS; and this effect of IL-10 was concentration-dependent (Figure 3a1,a2). In VM neuron-enriched cultures, LPS upregulated TNF-α mRNA and protein expression as well as COX-2 mRNA expression; and IL-10 prevented LPS-induced TNF-α upregulation but did not significantly affect COX-2 expression (Figure 3b1,b2). Other two pro-inflammatory mediators (iNOS and IL-1β) were not detected for their expression in VM neuron-enriched cultures. In both neuron-microglia co-cultures (Figure 3c1,c2) and neuron-astrocyte co-cultures (Figure 3d1,d2), LPS upregulated mRNA and protein expression of iNOS, COX-2, IL-1β and TNF-α; and IL-10 reduced the upregulated expression of the pro-inflammatory mediators induced by LPS. In addition, IL-10 exposure alone to any of the four different cell-composition cultures did not strikingly affect expression of the pro-inflammatory mediators (Figure 3).

### 2.4. IL-10 Prevents LPS-Induced Downregulation of Neurotrophic Factors in VM Cultures, VM Neuron-Enriched Cultures, or Neuron-Microglia Co-Cultures

**Figure 4:**
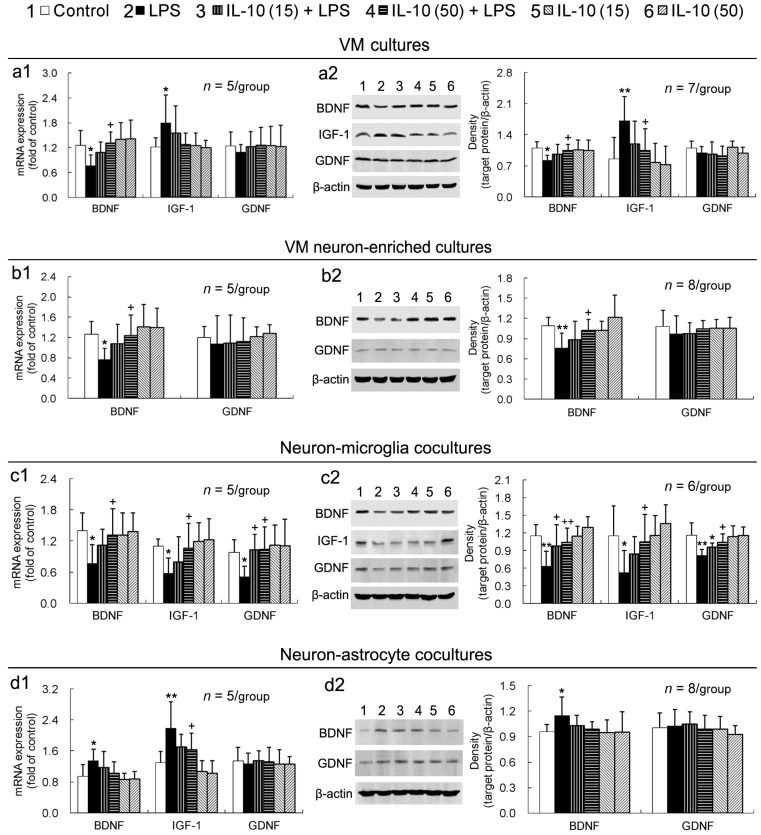
IL-10 prevents LPS-induced downregulation of neurotrophic factors in VM cultures, VM neuron-enriched cultures, or neuron-microglia co-cultures. The cultures were pretreated with IL-10 (15 or 50 ng/mL) for 1 h and then LPS (50 ng/mL) was added to the cultures, which were incubated for 24 h. Expression of genes (**a1**,**b1**,**c1**,**d1**) and proteins (**a2**,**b2**,**c2**,**d2**) of the neurotrophic factors was measured by real-time PCR and Western blot assay, respectively.* *p* < 0.05, ** *p* < 0.01, *versus* control; ^+^ *p* < 0.05, ^++^ *p* < 0.01 *versus* LPS. IL-10 (15): IL-10 (15 ng/mL); IL-10 (50): IL-10 (50 ng/mL).

LPS downregulated gene and protein expression of neurotrophic factor BDNF, but not GDNF, in VM cultures (Figure 4a1,a2). A higher concentration of IL-10 (50 ng/mL) prevented LPS-induced BDNF downregulation (Figure 4a1,a2). Interestingly, IGF-1, also a neurotrophic factor, was upregulated by LPS in VM cultures, and the upregulated IGF-1 was prevented by 50 ng/mL of IL-10 (Figure 4a1,a2). Similarly, in VM neuron-enriched cultures, LPS downregulated expression of BDNF (but not GDNF), and a higher concentration of IL-10 (50 ng/mL) prevented LPS-induced BDNF downregulation (Figure 4b1,b2). Dissimilarly, IGF-1 was not detected for the expression in VM neuron-enriched cultures. In neuron–microglia co-cultures, all the three examined neurotrophic factors, BDNF, IGF-1 and GDNF, were downregulated by LPS, and IL-10 (15 or 50 ng/mL) prevented LPS-induced downregulation of the neurotrophic factors (Figure 4c1,c2). In contrast, in neuron-astrocyte co-cultures, both BDNF and IGF-1 (but not GDNF) were upregulated by LPS (although IGF-1 protein expression was not detected), and IL-10 did not significantly alter LPS-induced BDNF upregulation (Figure 4d1,d2). Likewise, IL-10 exposure alone to any of the four different cell-composition cultures did not notably influence expression of the neurotrophic factors (Figure 4).

### 2.5. IL-10 Alleviates LPS-Induced Disorders in Releasing Pro-Inflammatory and Neurotrophic Factors in the Various Cell-Composition Cultures 

**Figure 5:**
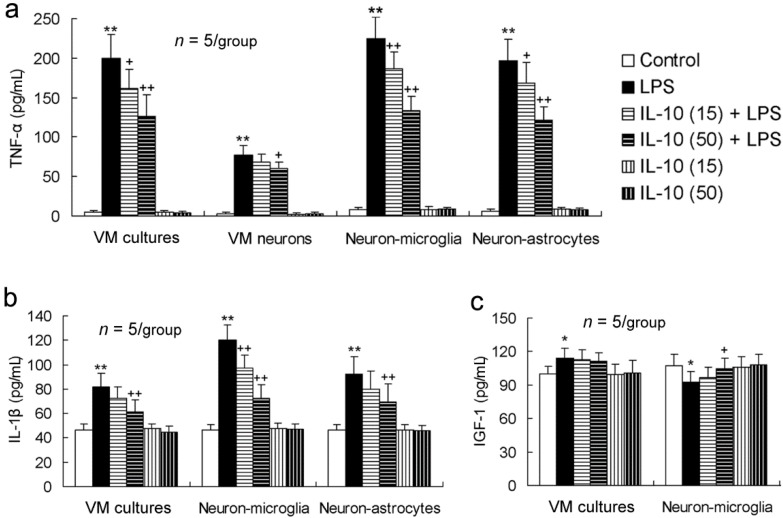
IL-10 alleviates LPS-induced disorders in releasing pro-inflammatory and neurotrophic factors in the various cell-composition cultures. The cultures were pretreated with IL-10 (15 or 50 ng/mL) for 1 h and then LPS (50 ng/mL) was added to the cultures, which were incubated for 24 h. The supernatants of the various cell-composition cultures were collected and detected by ELISA. * *p* < 0.05, ** *p* < 0.01, *versus* control; ^+^ *p* < 0.05, ^++^ *p* < 0.01 *versus* LPS. IL-10 (15): IL-10 (15 ng/mL); IL-10 (50): IL-10 (50 ng/mL).

To better show the anti-inflammatory property of IL-10, we determined the levels of pro-inflammatory cytokines TNF-α and IL-1β as well as neurotrophic factor IGF-1 in the supernatants of VM cultures, VM neuron-enriched cultures, neuron-microglia co-cultures and neuron-astrocyte co-cultures. As shown in mRNA and protein expression, LPS induced a striking increase in releasing TNF-α and IL-1β, and IL-10 significantly reduced this effect of LPS in the various cells (Figure 5a,b). IGF-1 release was also increased by LPS in VM cultures, similar to its expression, but IL-10 did not significantly alter LPS-induced IGF-1 release (Figure 5c). However, in neuron-microglia co-cultures, IGF-1 release was decreased by LPS, and a higher concentration of IL-10 (50 ng/mL) prevented this effect of LPS (Figure 5c). The data were consistent with those in IGF-1 mRNA and protein expression.

## 3. Discussion

LPS has been the most extensively utilized glial activator for the induction of inflammatory dopaminergic neurodegeneration [2]. In the present study, we found that LPS of 50 to 50,000 ng/mL (but not 5 ng/mL) induced both dopaminergic and non-dopaminergic neuronal loss in single neuronal cultures in a concentration-dependent manner, demonstrating a direct damage of LPS to neurons. Several earlier reports present that LPS is toxic to dopaminergic neurons only in the presence of microglia [32,33]. Recently, it has been suggested that LPS has a direct injury to hippocampal CA1 pyramidal neurons [34,35]. Here, we provide further evidence showing that LPS directly induces dopaminergic and non-dopaminergic neuronal death. The damage not limited to dopaminergic neurons by LPS is supported by the results that intraperitoneal injections of LPS induce intracellular accumulation of Aβ_1–42_ in hippocampal pyramidal neurons [36,37]. However, others report that a single intranigral injection of LPS induces an acute inflammatory response with a strong microglial reaction, producing a selective death of dopaminergic but not GABAergic neurons in the substantia nigra [38,39]. A possible explanation for the phenomena of LPS selective or non-selective injury to dopaminergic neurons is attributed to the different regions, ranges, degree and duration influenced by LPS in the brain and the different objects observed *in vitro*. Importantly, we found astrocytic activation by LPS does not produce significant toxicity to either dopaminergic or non-dopaminergic neurons. This is because astrocytes produce more neurotrophic factors that can antagonize the increased pro-inflammatory mediators in response to LPS stimulation. Indeed, microglia have been identified as the major LPS responsive cells in the brain [40]. Particularly in LPS-induced PD model, microglia play a more prominent role than astrocytes in the release of various neurotoxic factors that cause dopaminergic neurodegeneration [41]. Even so, astrocytes are still used as the cells of inflammatory response to LPS, producing inflammatory mediators that may injure neurons [42,43]. In the current study, we propose that astrocytic activation induced by LPS is a double-edged sword: it may represent protective mechanisms by releasing neurotrophic factors and also may contribute to neuronal damage by producing pro-inflammatory mediators, depending on pathological conditions and tissue-injured degree.

Notably, IL-10 reduced LPS-induced dopaminergic and non-dopaminergic neuronal loss in various cell-composition cultures, confirming a neuroprotective property of this cytokine. In particular, IL-10 neuroprotection occurring in single neuronal cultures suggests a direct effect of IL-10 on neurons. As a potent anti-inflammatory cytokine, IL-10 is mostly reported to exert neuroprotective property by inhibiting microglial activation and inflammatory responses [30,44,45,46]. However, though a few, the reports have indicated that neurons express IL-10 receptors and via the receptors, IL-10 exerts a direct trophic influence on spinal cord neurons and increases neuronal survival in cortical neuron cultures after exposure to glutamate toxicity [47,48]. Here we for the first time show that IL-10 neuroprotection against LPS is not necessarily dependent on its inhibition of microglial inflammatory responses. 

On the other hand, since microglia are the major players in the inflammatory processes that mediate LPS-induced neurotoxicity [49,50,51,52], inhibiting microglial over-activation may bring an evident protection of neurons. As shown in this study, LPS (5 or 50 to 50,000 ng/mL) strikingly induced NO and H_2_O_2_ release in VM cultures in a concentration-dependent manner, demonstrating that LPS induces a glial oxidative stress state. The release of H_2_O_2_ by microglia has been found to precede dopaminergic neuronal death [53]. The glial-derived RNS and ROS can harm nearby neurons and ultimately result in neuronal cell death [54]. Significantly, IL-10 reduced the NO and H_2_O_2_ release induced by LPS in this study. This suggests that IL-10 has an antioxidant mechanism by which it implements the neuroprotection. Moreover, IL-10 inhibited both microglial and astrocytic inflammatory responses to LPS as determined by a decreased pro-inflammatory mediator production and an increased neurotrophic factor production. These properties of IL-10 enhance its anti-inflammatory and neuroprotective effects. Collectively, IL-10 exerts neuroprotective property by an extensive action including direct on neurons and indirect via microglia and astrocytes.

## 4. Experimental Section

### 4.1. Cell Culture

#### 4.1.1. Primary VM Cultures

VM cultures were prepared from the VM tissues of embryonic Day 14 (E14) ± 0.5 Sprague-Dawley rats (Center of Experimental Animals, Nantong University, Nantong, China), as described previously [49,55]. In brief, VM tissues were removed from fetuses and dissociated to single cells by a mechano-enzymatic method involving a protease treatment with 2.5 mg/mL trypsin (Amresco, Solon, OH, USA) and additional mechanical shearing. After centrifugation, cells were suspended in Dulbecco’s modified Eagle’s medium/nutrient mixture F-12 (DMEM/F12, Invitrogen, Carlsbad, CA, USA) supplemented with 10% FBS and seeded at a density of 5 × 10^5^ cells/cm^2^ on 24- or 6-well plates precoated with 0.01% poly-l-lysine (sigma-Aldrich, St. Louis, MO, USA), respectively. Cells were incubated at 37 °C with a humidified atmosphere of 5% CO_2_ and 95% air. Seven-day-old cultures were used for treatments.

#### 4.1.2. Primary VM Neuron-Enriched Cultures

Dissociated rat VM cells were seeded first at 5 × 10^5^ cells/cm^2^ onto 24- or 6-well culture plates precoated with poly-l-lysine as described above. On the second day after initial seeding, cytosine β-d-arabinofuranoside (5–10 μM) was added to suppress glial proliferation. After incubated for 2 or 3 days, the cultures were changed back to fresh medium. Those seven-day-old cultures that contained about 95% NeuN-IR neurons were used for treatments.

#### 4.1.3. Primary Neuron-Microglia Co-Cultures

Primary microglia were isolated from newborn Sprague-Dawley rats according to a previous study [49], with some modifications. Briefly, cerebral cortices of one-day-old Sprague-Dawley rat pups, with the blood vessels and meninges removed, were chemically dissociated in the presence of trypsin at 37 °C for 15 min. The isolated cells (1 × 10^7^) were seeded in 25 cm^2^ culture flasks in DMEM/F12 containing 10% FBS and incubated for 12–14 days to obtain mixed glial cells. After reaching confluence, microglia were harvested from the mixed glial cells by shaking the flasks at 200 rpm for 4 h on a rotary shaker. After centrifugation, the cells were resuspended in fresh medium, and seeded onto the plates as enriched microglia for further studies. About 90%–95% of this preparation was found to be positive for CD11b surface antigen, a marker for microglia [56].

Reconstituted cell cultures were prepared as described previously [57]. VM neuron-enriched cultures were first prepared as mentioned above. On the sixth day after the initial seeding, the highly enriched microglia (5 × 10^4^ cells/cm^2^) were added to neuron-enriched cultures. One day later, the reconstituted cell cultures were treated with LPS.

#### 4.1.4. Primary Neuron-Astrocyte Co-Cultures

Cerebral cortices were processed into glial cell cultures as described above. To obtain primary astrocytes, the remnant cells after the separation of microglia were detached with trypsin-EDTA, which were seeded in the same culture medium as used for microglia. After at least four consecutive passages, almost pure (no more than 2% contamination) astrocytic cell populations were obtained [58].

Neuron-astrocyte co-cultures were prepared by coculturing fetal VM neurons with the nearly pure astrocytes (1 × 10^5^ cells/cm^2^) on the sixth day after the fetal VM neurons from E14 ± 0.5 rats were cultured. One day later, the neuron-astrocyte co-cultures were treated with LPS.

### 4.2. Drug Exposures

In the primary VM cultures or VM neuron-enriched cultures, recombinant rat IL-10 (Peprotech, London, UK) was applied on day 7 after the cultures were incubated at the concentrations of 15, 50, or 150 ng/mL, respectively, on the basis of the reports [47,59,60,61]. LPS (*Escherichia coli* 0111:B4, Sigma-Aldrich, St. Louis, MO, USA) was added 1 h following IL-10 application at a concentration of 50 ng/mL, as described by Fahlenkamp *et al*. [62] and Shi *et al*. [63]. In the primary neuron–microglia or neuron-astrocyte co-cultures, IL-10 was applied at 24 h after the co-cultures were incubated, which was treated with LPS in the same manner as above. They were incubated for 24 h and then assessed for dopaminergic neuronal loss and inflammatory responses.

### 4.3. Immunocytochemistry

After stimulation with LPS for 24 h, the cells grown on the coverslipes inside the 24-well plates were fixed for 20 min at room temperature in formaldehyde (4%), followed by sequential incubation with blocking solution for 30 min, primary antibodies against neuron-specific nuclear protein (NeuN, 1:300, Millipore, Billerica, MA, USA) and TH (1:300, Millipore) overnight at 4 °C, and appropriate fluorescent secondary antibodies for 6 h at room temperature. The immunoreactive cells were viewed and counted under a fluorescence microscope (Leica, Wetzlar, Germany) at 20× magnification. Number of TH^+^NeuN^+^ cells in 25 visual fields was reported as sum for each coverslip, and number of TH^–^NeuN^+^ cells was counted in 5 visual fields and reported as average for each coverslip.

### 4.4. NO Assay

NO production was assessed by the accumulation of nitrite in the culture supernatants using a colorimetric reaction with Griess reagent following manufacture’s protocol. Briefly, the supernatants were collected at 24 h after the cultures were stimulated with LPS and mixed with an equal volumes of Griess reagent (0.1% *N*-(1-naphthyl) ethylenediamine dihydrochloride, 1% sulfanilamide, and 2.5% H_3_PO_4_) at room temperature for 10 min. The absorbance at 540 nm was measured with an ultraviolet MAX kinetic microplate reader (Molecular Devices, Menlo Park, CA, USA). The nitrite concentration was determined from a sodium nitrite standard curve.

### 4.5. H_2_O_2_ Assay

H_2_O_2_ analysis was carried out using an H_2_O_2_ assay kit (Beyotime Biotech, Shanghai, China) as described previously [64,65]. H_2_O_2_ oxidizes Fe^2+^into Fe^3+^, and then Fe^3+^ reacts with xylenol orange resulting in a colorimetric reaction that can be detected by a spectrometer. Briefly, test tubes containing 50 μL of culture supernatants and 100 μL of test solutions were placed at room temperature for 20 min and then measured immediately by a spectrometer at a wavelength of 560 nm. The concentration of H_2_O_2_ released was calculated by a standard concentration curve.

### 4.6. Real-Time Reverse Transcription-PCR Analysis

At 24 h after LPS treatment, total RNA of different cell-component cultures was extracted by Trizol reagent (Invitrogen) according to the manufacturer’s instructions. Potentially contaminating residual genomic DNA was eliminated with RNAse-free DNAse (Promega, Madison, WI, USA). cDNA was synthesized from 2 μg of RNA using reverse transcriptase (Promega) with oligo (dT) primer (Invitrogen) in a 20 μL reaction. We quantified the PCR amplifications using the Rotor-Gene 3000 Real-time Cycler (Corbett Research, Sydney, Australia) with SYBR green I (Molecular Probe, Eugene, OR, USA) as the detection system. The data were collected using the instrument’s software (Rotor-Gene software, version 6.0, Corbett Research, Sydney, Australia) and relative quantification was performed using the comparative threshold (*C*_t_) method after determining the *C*_t_ values for reference (*β-actin*) and target genes (*iNOS*, *COX-2*, *TNF-α*, *IL-1β*, *GDNF*, *BDNF* or *IGF-1*) in each sample set according to the 2^−ΔΔ*C*t^ method. Changes in mRNA expression levels were calculated after normalization to *β-actin*. The primer sequences of the target genes are listed in Table 1.

**Table 1:** Sequences of PCR primers.

Gene	Sense Primer (5′-3′)	Antisense Primer (5′-3′)
*iNOS*	CAGCTGGGCTGTACAAACCTT	CATTGGAAGTGAAGCGTTTCG
*COX-2*	CCAGCAGGCTCATACTGATAGGA	GCAGGTCTGGGTCGAACTTG
*TNF-α*	CCACCACGCTCTTCTGTCTAC	ATCTGAGTGTGGGGTCTGG
*IL-1β*	CTTCCTTGTGCAAGTGTCTG	CAGGTCATTCTCATCACTGTC
*GDNF*	ATTCAAGCCACCATCAAAAG	TCAGTTCCTCCTTGGTTTCG
*BDNF*	ATCCCATGGGTTACACGAAGGAAG	AGTAAGGGCCCGAACATACGATTG
*IGF-1*	TTTTACTTCAACAAGCCCACA	CATCCACAATGCCCGTCT
*β-actin*	CGTTGACATCCGTAAAGACC	TAGAGCCACCAATCCACAC

### 4.7. Western Blot Analysis

The various cultured cells grown in 6-well plates were harvested and homogenized in lysis buffer following LPS treatment. The cellular proteins were separated by 10% or 12% sodium dodecyl sulfate-polyacrylamide gel electrophoresis (SDS-PAGE) and transferred to polyvinylidene difluoride membranes (Pall, Port Washington, NY, USA) using an electroblotting apparatus (Bio-Rad, Hercules, CA, USA). The membranes were probed with the primary antibodies to COX-2 (1:200, Santa Cruz Biotechnology, Inc., Santa Cruz, CA, USA), GDNF (1:200, Santa Cruz Biotechnology), BDNF (1:200, Santa Cruz Biotechnology), IL-1β (1:200, Santa Cruz Biotechnology), IGF-1 (1:200, Santa Cruz Biotechnology), iNOS (1:300, Abcam, Cambridge, UK) and TNF-α (1:300, Abcam). Monoclonal anti-β-actin antibody (Sigma-Aldrich) was included as an internal standard to monitor loading errors. Appropriate IRDye 800-conjugated secondary antibodies (1:5000, Rockland Immunochemicals, Inc., Gilbertsville, PA, USA) were used to visualize blots using Odyssey laser scanning system (LI-COR Inc., Lincoln, NE, USA). The molecular weight and relative quantity of the protein bands were determined by an image analysis system (Odyssey 3.0 software, LI-COR Inc., Lincoln, NE, USA).

### 4.8. Determination of Cytokine and Neurotrophic Factor Levels by Enzyme-Linked Immunosorbent Assay (ELISA)

Concentrations ofthe pro-inflammatory cytokines, TNF-α and IL-1β, and the neurotrophic factor, IGF-1, in supernatants of various cell-composition cultures were measured using ELISA kits (eBioscience, San Diego, CA, USA, for TNF-α and IL-1β, and Abcam, UK, for IGF-1) according to the manufacturers’ protocol. The cytokine and neurotrophic factor concentrations in the test samples were evaluated with reference to standard curve prepared using recombinant cytokines and neurotrophic factor of known concentrations.

### 4.9. Statistical Analysis

Statistical analyses were performed with the Statistics Package for Social Science (SPSS, 13.0, SPSS, Chicago, IL, USA). Data were presented as mean ± standard deviation (M ± SD). The difference between means was determined by the one-way analysis of variance (ANOVA) followed by a Student-Newman-Keul’s test to compare the data of all groups relative to each other. Differences were considered statistically significant at *p* < 0.05.

## 5. Conclusions

LPS induced both dopaminergic and non-dopaminergic neuronal loss in VM cultures, VM neuron-enriched cultures, and neuron-microglia co-cultures, but not in neuron-astrocyte co-cultures. These data indicate that LPS has a direct toxicity to neurons and that astrocytes can balance LPS neurotoxicity. As expected, the pro-inflammatory mediators, iNOS, COX-2, IL-1β and TNF-α, were upregulated, but the neurotrophic factors, BDNF, IGF-1 and GDNF, were downregulated by LPS in the presence of microglia, supporting an over-activation of microglia in response to LPS stimulation. In contrast, LPS induced BDNF and IGF-1 upregulation in the presence of astrocytes, although it also induced pro-inflammatory mediator upregulation. These phenomena suggest that astrocytic activation is a double-edged sword that can neutralize LPS neurotoxicity. Importantly, IL-10 reduced LPS-induced dopaminergic and non-dopaminergic neuronal loss particularly in the single VM neuronal cultures, showing that IL-10 has a direct protective effect on neurons. In addition, IL-10 inhibited pro-inflammatory mediators but elevated neurotrophic factors in the presence of LPS, demonstrating the anti-inflammatory and neuroprotective properties of this cytokine. The properties of IL-10 make it promising to become a candidate for treatment of inflammatory neurodegenerative diseases including PD.
